# Interval changes in ROTEM values during cardiopulmonary bypass in pediatric cardiac surgery patients

**DOI:** 10.1186/s13019-019-0949-0

**Published:** 2019-07-22

**Authors:** Christopher F. Tirotta, Richard G. Lagueruela, Daria Salyakina, Weize Wang, Thomas Taylor, Jorge Ojito, Kathleen Kubes, Hyunsoo Lim, Robert Hannan, Redmond Burke

**Affiliations:** 1 Department of Anesthesia, The Heart Program, Nicklaus Children’s Hospital, 3100 S.W. 62nd Street, Miami, FL 33155 USA; 2 Research Institute, Nicklaus Children’s Hospital, Miami, FL USA; 3 Department of Cardiac Surgery, The Heart Program, Nicklaus Children’s Hospital, 3100 S.W. 62nd Street, Miami, FL 33155 USA

**Keywords:** ROTEM, Fibrinogen, RiaSTAP, Neonates, Infants, Cardiac surgery

## Abstract

**Introduction:**

Rotational thromboelastometry (ROTEM) has been shown to reduce the need for transfused blood products in adult and pediatric cardiac surgery patients. However, similar evidence in newborns, neonates, and young infants is lacking. We quantified ROTEM value changes in pediatric patients on cardiopulmonary bypass (CPB) before, during and after blood product transfusion.

**Methods:**

Each surgery had at least four interventions: initiating CPB; platelet administration during rewarming phase; post-CPB and following protamine and human fibrinogen concentrate (HFC) administration; and further component therapy if bleeding persisted and ROTEM indicated a deficiency. ROTEM assays were performed prior to surgery commencement, on CPB prior to platelet administration and following 38 mL/kg platelets, and post-CPB after protamine and HFC administration. ROTEM assays were also performed in the post-CPB period after further blood component therapy administration.

**Results:**

Data from 161 patients were analyzed. Regression models suggested significant changes in HEPTEM clotting time after all interventions. PLT administration during CPB improved HEPTEM α by 22.1° (*p* < 0.001) and FIBTEM maximum clot firmness (MCF) by 2.9 mm (p < 0.001). HFC administration after CPB termination significantly improved FIBTEM MCF by 2.6 mm (p < 0.001). HEPTEM MCF significantly increased after 3/4 interventions. HEPTEM α significantly decreased after two interventions and significantly increased after two interventions. Greatest perturbances in coagulation parameters occurred in patients ≤90 days of age.

**Conclusion:**

CPB induced profound perturbations in ROTEM values in pediatric cardiac surgery patients. ROTEM values improved following PLT and HFC administration. This study provides important clinical insights into ROTEM changes in pediatric patients after distinct interventions.

## Introduction

Rotational thromboelastometry (ROTEM, Tem International GmbH, Munich, Germany) is an enhanced modification of thromboelastography (TEG, Haemonetics Corp., Braintree, MA, USA), first described in 1948 [1]. Both are point-of-care (POC) coagulation monitoring instruments that test the viscoelastic properties of whole blood [1]. TEG and ROTEM, while similar, have several differences which can lead to discrepancies in results obtained, particularly for fibrin-based clotting measurements [2, 3].

A series of assays are employed with the ROTEM instrument. EXTEM provides information on the coagulation process via the extrinsic pathway and its interaction with thrombocytes in citrated blood; the reagent contains tissue factor and phospholipids used for extrinsic activation. HEPTEM provides information on the coagulation process via the intrinsic pathway in the presence of unfractionated heparin; this assay is similar to INTEM with the addition of heparinase to inactivate in vitro heparin. FIBTEM provides information on the fibrinogen level and quality of fibrin polymerization in citrated blood by inhibiting thrombocytes; the reagent contains thrombocyte inhibitor and recalcification reagent. APTEM provides information on clot firmness by inhibiting hyperfibrinolysis with aprotinin.

ROTEM has previously been validated for bedside use, with no significant differences in results compared with traditional laboratory assays, but a mean time saving of 11 (8–16) minutes [4]. A prospective study by Ogawa et al compared values obtained using standard laboratory coagulation tests with ROTEM values in adult patients undergoing cardiac surgery, demonstrating that some ROTEM measurements could act as surrogates for standard coagulation tests [5]. However, although reference ROTEM values in pediatric patients have been described [6], similar data are lacking in newborns, neonates and young infants. The coagulation system functions very differently in these patient groups compared with older children and adults.

The aim of this study is to define and quantify how cardiopulmonary bypass (CPB) transfusion therapy with human fibrinogen concentrate (HFC), plateletpheresis (PLT), and other blood products affect ROTEM values.

## Methods

### Study design

A retrospective analysis of pediatric patients undergoing cardiac surgery requiring CPB at Nicklaus Children’s Hospital, Miami, FL, USA between June 1, 2015 and August 31, 2017. This study received Institutional Review Board (IRB) exempt status from the Research Institute of Nicklaus Children’s Hospital.

For each assay, the following parameters were considered: clotting time (s) (CT; time to reach 2 mm of amplitude from the test initiation); alpha (α; angle of the line between horizontal and the line from CT point to CFT point); clot formation time (s) (CFT; time between reaching 2 mm amplitude and 20 mm amplitude) and maximum clot firmness (mm) (MCF; maximum amplitude).

In accordance with standard institutional protocol, ROTEM assays were performed at the following times:

- ROTEM 1: baseline value obtained at the start of surgery prior to CPB initiation.
- ROTEM 2: during the rewarming phase of CPB, prior to PLT administration (occurring after the administration of blood products in the priming phase).
- ROTEM 3: on CPB after PLT administration.
- ROTEM 4: after CPB termination, and after protamine and HFC administration.
- ROTEM 5: after the administration of other blood products if bleeding persisted and ROTEM 4 indicated a deficiency.

Each surgery had at least four interventions.

- Intervention I: when initiating CPB, for patients less than 10 kg, the pump was primed with 1 U PRBC. If the patient was less than 3 kg, 50 mL of PLT were added to the prime. If the patient was antithrombin III deficient or the heparin management system revealed heparin resistance (based on pre-operative values), 60 mL of fresh frozen plasma (FFP) was added to the prime.
- Intervention II: administration of PLT given during the rewarming phase of CPB with a median dose of 38 mL/kg.
- Intervention III: administration of Protamine, and HFC (70 mg/kg; RiaSTAP, CSL Behring, Marburg, Germany) after CPB termination.
- Intervention IV: further component therapy if bleeding persisted after the HFC and ROTEM indicated a deficiency. This could be further HFC, PLT, FFP or cryoprecipitate

### Statistical analysis methods

Descriptive statistics, including sample median and interquartile range, were calculated for patients’ characteristics at baseline and at the four intervention time points, for blood or coagulation products (packed red blood cells [PRBC], platelets, FFP, Cell Saver [CS], Cryoprecipitate, phlebotomized blood [PB], or HFC). Calculations were performed for both the overall population and subgroups stratified by age (≤90 days; > 90 days and ≤ 2 years of age; and > 2 years of age). At intervention I, PRBC and FFP could be provided twice, so the cumulative dose for each was used when calculating medians and interquartile ranges. Kruskal-Wallis/Mann Whitney U tests were performed to identify any significant difference in the distribution of each variable by age groups.

Regression analysis was performed to adjust for the variation in per patient blood product administration. Generalized estimation equation (GEE) modeling was applied to predict HEPTEM CT, HEPTEM MCF and FIBTEM MCF (when data were available) to quantify the change in ROTEM values after each wave of platelets/HFC administration. Generalized linear mixed modeling with a beta distribution was employed to model the proportion of HEPTEM α in the 0–90° range. Proportions estimated by the model were then rescaled to the original scale of measurement (0–90°) for ease of interpretation of regression results.

Predictors included in the GEE and beta regression models were age group, before/after platelet or HFC administration, administration of each of the blood products, and the interaction between age group and the before/after platelet or HFC administration predictors.

Results of HEPTEM CT > 1800 s were excluded from regression analysis due to outliers affecting the assumptions and convergence of the GEE models. Predicted marginal means (SAS PROC LSMEANS) with 95% confidence intervals (CIs) of ROTEM values (HEPTEM CT, HEPTEM MCF, FIBTEM MCF, and HEPTEM α) were calculated by age group for timepoints before and after each wave of administration, and the change between timepoints. Bonferroni-corrected *p*-values were calculated for the changes in ROTEM values by age group.

All data analysis was performed at a 0.05 level of significance and conducted using SAS Enterprise Guide 7.1 (SAS Institute, Cary, NC, USA).

## Results

A total of 161 pediatric patients (median age, 214 days; interquartile range, 1324 days) were included in the study. Of these patients, 26% were ≤ 90 days of age, 40% were between 90 days and 2 years of age, and 34% were older than 2 years. Table 1 presents descriptive statistics and comparison between the three age groups. Some parameters varied significantly between these age groups during the surgery. The median bypass time was the longest for youngest patients (146.5 min), and shortest in the > 90 days ≤2 years group (87.0 min; *p* < 0.001). The youngest patients also had the lowest median low temperature during the surgery, relative to the two older groups (p < 0.001). Because of the very small sample size, there were no statistically significant differences between the three groups in cross clamp time, incidence of regional low flow perfusion or deep hypothermic circulatory arrest; however, there was a tendency towards higher median values in younger children.

**Table 1:** Descriptive statistics of patients’ characteristics and interventions (*N* = 161)

	Overall			Age Groups									p^b^
				90 days or less			Older than 90 days to 2 years			Older than 2 years			
	N^a^	Median	IQR	N	Median	IQR	N	Median	IQR	N	Median	IQR	
Weight (kg)	161	6.4	12.0	42	3.2	0.8	64	6.2	2.8	55	24.0	38.0	**< 0.001**
Height (cm)	161	65.0	47.0	42	50.3	5.0	64	64.0	10.3	55	127.0	57.0	**< 0.001**
Age (days)	161	214.0	1324.0	42	9.5	38.0	64	193.5	147.5	55	2649.0	3582.0	**< 0.001**
Bypass (min)	161	106.0	74.0	42	146.5	64.0	64	87.0	46.0	55	106.0	73.0	**< 0.001**
Cross clamp time (min)	140	62.0	49.5	42	82.5	56.0	52	55.5	38.0	46	58.5	46.0	0.053
Regional low flow perfusion (min)	24	102.0	66.5	22	114.5	57.0	2	31.5	55.0	0	0.0	0.0	0.089
Deep hypothermic circulatory arrest (min)	15	15.0	41.0	12	9.5	38.0	3	27.0	43.0	0	0.0	0.0	0.829
Low temperature (c)	159	29.1	7.2	42	17.8	7.8	64	29.4	3.3	53	30.1	4.6	**< 0.001**
Intervention I													
Packed red blood cells (mL/kg)	108	62.0	52.0	42	96.5	77.0	62	44.5	23.0	4	13.5	14.5	**< 0.001**
Plateletpheresis (mL/kg)	13	23.0	8.0	10	22.0	8.0	3	26.0	12.0	0	0.0	0.0	0.934
Fresh frozen plasma (mL/kg)	27	24.0	9.0	21	23.0	7.0	5	26.0	8.0	1	6.0	0.0	0.055
Intervention II													
Plateletpheresis (mL/kg)	95	38.0	29.0	40	56.0	17.5	54	29.5	12.0	1	18.0	0.0	**< 0.001**
Intervention III													
HFC (mg/kg)	129	70.0	2.0	40	70.0	1.0	58	70.0	2.0	31	68.0	7.0	**< 0.001**
Intervention IV													
HFC (mg/kg)	45	70.0	1.0	24	70.0	1.0	19	70.0	1.0	2	46.5	47.0	0.229
Plateletpheresis (mL/kg)	81	13.0	11.0	40	19.0	13.5	40	9.0	3.5	1	5.0	0.0	**< 0.001**
Cell saver (mL/kg)	151	17.0	19.0	36	33.0	22.0	62	19.0	9.0	53	5.0	4.0	**< 0.001**
Fresh frozen plasma (mL/kg)	22	19.5	13.0	17	20.0	9.0	4	15.5	51.5	1	3.0	0.0	0.226
Packed red blood cells (mL/kg)	18	20.5	22.0	12	25.0	13.0	4	6.5	10.0	2	8.0	6.0	**0.008**
Cryoprecipitate (mL/kg)	2	10.0	0.0	1	10.0	0.0	1	10.0	0.0	0	0.0	0.0	NA
Phlebotomized blood (mL/kg)	19	10.0	4.0	0	0.0	0.0	0	0.0	0.0	19	10.0	4.0	NA

^a^N is number of patients received the procedure/blood product. Descriptive statistics excluded patients who did not receive the procedure/product

^b^*p* values from Kruskal Wallis test by age groups. When there was no patient in the age group, Mann Whitney U test was applied. If p = NA (Not Available), sample size was too small to perform the test

*HFC* human fibrinogen concentrate, *IQR* interquartile range

*p* values in bold represent statistical significance

**Fig. 1:**
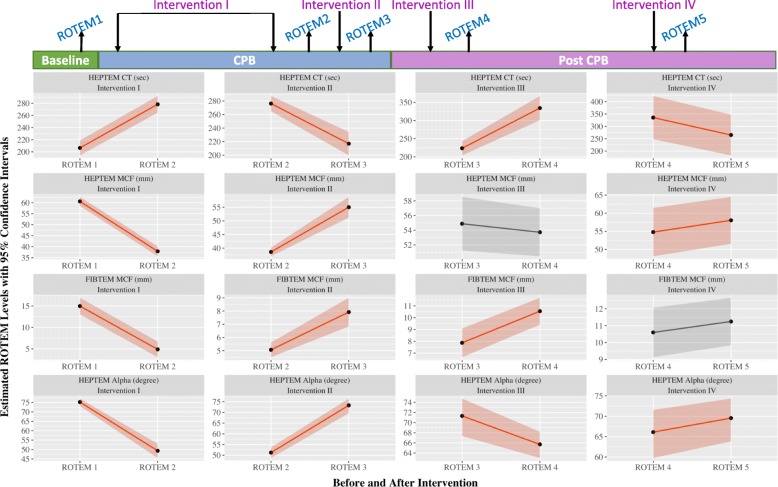
Estimated ROTEM values before and after interventions. Intervention means each waves of administration of blood products. CPB pre-platelets were prime CPB PRBC, prime CPB platelets and prime CPB FFP. CPB post-platelets included run CPB PRBC, run CPB platelets and run CPB FFP. Post bypass HFC was HFC only. Post-bypass platelets included HFC, platelets, CS, FFP, PRBC, Cryo and PB. Estimates before and after each intervention were predicted marginal means. Significant changes in ROTEM levels were marked in red. CPB, cardiopulmonary bypass; CS, cell saver; CT, clotting time; FFP, fresh frozen plasma; HFC, human fibrinogen concentrate; MCF, maximum clot firmness; PB, phlebotomized blood; PRBC, packed red blood cells

Results from GEE and beta regression models suggested significant overall changes in several intervention waves for all four ROTEM measurements (Fig. 1, Table 2). Specifically, significant changes were observed in HEPTEM CT and HEPTEM α after all four interventions; and HEPTEM MCF and FIBTEM MCF after three interventions. Moreover, mean fibrinogen levels decreased 162.1 mg/dL (range 136.5–187.7 mg/dL, *p* < 0.001) after Intervention I, increased 69.8 mg/dL (range 58.3–81.2 mg/dL, *p* < 0.001) after Intervention II, and increased 73.1 mg /dL (range 43.1–103.1, *p* = .001) after Intervention III.

**Table 2:** Estimated changes in ROTEM values and fibrinogen levels after four waves of blood product transfusion (intervention)

Outcome	Intervention	Estimates				p^b^
		N^a^	Before (95% CI)	After (95% CI)	Change (95% CI)	
HEPTEM CT (sec)	Intervention I	266	206.5 (194.2, 218.9)	278.4 (264.6, 292.2)	71.9 (59.8, 83.9)	**< 0.001**
	Intervention II	206	276.2 (264.9, 287.4)	217.0 (199.7, 234.4)	−59.1 (−79.3, −39.0)	**< 0.001**
	Intervention III	219	223.7 (204.0, 243.4)	334.4 (301.1, 367.8)	110.8 (77.7, 143.8)	**< 0.001**
	Intervention IV	189	335.6 (248.1, 423.1)	265.1 (182.5, 347.7)	−70.5 (−102.8, −38.2)	**< 0.001**
HEPTEM MCF (mm)	Intervention I	266	60.6 (58.5, 62.7)	37.9 (35.6, 40.2)	−22.7 (−24.2, −21.2)	**< 0.001**
	Intervention II	205	38.6 (37.0, 40.2)	55.0 (51.2, 58.8)	16.4 (12.4, 20.4)	**< 0.001**
	Intervention III	217	54.9 (51.2, 58.5)	53.7 (50.4, 57.0)	−1.2 (−3.7, 1.4)	0.380
	Intervention IV	188	54.8 (48.1, 61.5)	58.0 (51.5, 64.5)	3.3 (1.1, 5.4)	**0.003**
FIBTEM MCF (mm)	Intervention I	255	15.0 (13.0, 16.9)	4.9 (3.1, 6.7)	−10.1 (−11.0, −9.2)	**< 0.001**
	Intervention II	196	5.1 (4.5, 5.6)	7.9 (6.8, 9.0)	2.9 (1.8, 4.0)	**< 0.001**
	Intervention III	219	7.9 (6.7, 9.1)	10.5 (9.4, 11.7)	2.7 (2.0, 3.4)	**< 0.001**
	Intervention IV	190	10.6 (9.1, 12.1)	11.2 (9.8, 12.7)	0.6 (−0.3, 1.6)	0.197
HEPTEM α (°)	Intervention I	265	75.2 (73.1, 77.1)	49.3 (45.5, 53.1)	−25.9 (−30.8, −21.0)	**< 0.001**
	Intervention II	204	51.2 (48.7, 53.7)	73.3 (69.6, 76.5)	22.1 (16.8, 27.4)	**< 0.001**
	Intervention III	217	71.3 (67.4, 74.7)	65.7 (63.0, 68.2)	−5.6 (−8.5, −2.7)	**< 0.001**
	Intervention IV	188	66.1 (59.8, 71.6)	69.6 (63.8, 74.3)	3.4 (1.0, 5.9)	**0.005**

^a^N was number of observations used for each model. All the models were controlled for age groups, whether each of the interventions was received, as well as interaction between age groups and before or after the intervention

^b^Generalized linear mixed model using beta distribution was applied to predict α (°), while generalized estimation equation was used for other outcomes. Estimates were predicted marginal means

*CI* confidence interval, *CT* clotting time, *MCF* maximum clot firmness

*p* values in bold represent statistical significance

### Analysis of ROTEM parameters by age group

#### Intervention I: CBP priming and initiation

**Fig. 2:**
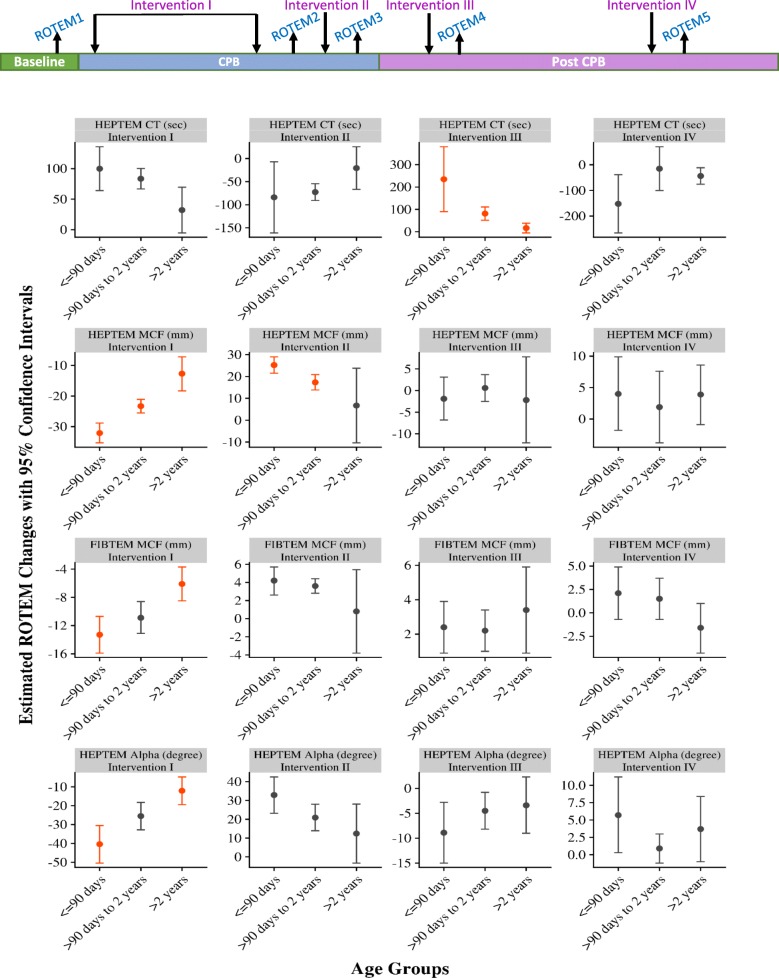
Estimated changes in ROTEM values by age groups. Estimated changes were predicted marginal means from generalized estimation equation models for HEPTEM MCF, HEPTEM CT, and FIBTEM MCF, and generalized linear mixed models for HEPTEM Alpha. A negative change indicates a decrease in the ROTEM value from before to after an intervention, whereas a positive change indicates an increase in the ROTEM value from before to after the intervention. The change is not significant if the 95% confidence interval crossed 0. Pairwise comparisons of changes in ROTEM values were performed by age groups using 95% confidence intervals. Age groups with significant difference were marked in red. CT, clotting time; MCF, maximum clot firmness.

A significant increase in HEPTEM CT was observed for patients aged ≤90 days (estimated mean change [EMC]: 99.9, 95% CI: 64.0–135.8, *p* < 0.001) and those > 90 days and ≤ 2 years (EMC: 83.5, 95% CI: 66.8–100.3, *p* < 0.001). There was no significant change in mean HEPTEM CT among patients > 2 years (*p* = 0.175, Table 3; Fig. 2).

**Table 3:** Estimated average changes in ROTEM values after four waves of interventions (blood product transfusions)

Outcome	Intervention	N^a^	Age Group	Estimated ROTEM Values			p^b,c^
				Before (95% CI)	After (95% CI)	Change (95% CI)	
HEPTEM CT (sec)	Intervention I	266	90 days or less	223.2 (202.0, 244.5)	323.1 (295.9, 350.4)	99.9 (64.0, 135.8)	**< 0.001**
	Intervention II	206		305.9 (282.3, 329.4)	221.8 (175.3, 268.3)	−84.0 (−161.1, −7.0)	**0.021**
	Intervention III	219		231.5 (185.4, 277.5)	466.3 (382.7, 550.0)	234.9 (89.9, 379.8)	**< 0.001**
	Intervention IV	189		444.2 (345.7, 542.7)	291.9 (221.9, 361.9)	−152.3 (−265.6, −39.0)	**0.001**
	Intervention I	266	Older than 90 days to 2 years	210.2 (187.5, 232.9)	293.7 (272.1, 315.3)	83.5 (66.8, 100.3)	**< 0.001**
	Intervention II	206		280.0 (266.9, 293.2)	207.3 (194.9, 219.7)	−72.7 (−90.8, −54.6)	**< 0.001**
	Intervention III	219		217.1 (199.7, 234.5)	298.1 (269.2, 327.0)	81.0 (51.1, 110.9)	**< 0.001**
	Intervention IV	189		294.8 (201.3, 388.2)	279.4 (176.5, 382.2)	−15.4 (−100.6, 69.8)	1.000
	Intervention I	266	Older than 2 years	186.1 (166.2, 206.1)	218.3 (191.9, 244.7)	32.2 (−5.3, 69.6)	0.175
	Intervention II	206		242.7 (219.9, 265.4)	207.3 (194.9, 219.7)	−20.7 (−66.7, 25.4)	1.000
	Intervention III	219		222.5 (204.7, 240.4)	238.9 (217.5, 260.3)	16.4 (−5.3, 38.1)	0.401
	Intervention IV	189		267.8 (159.1, 376.4)	223.9 (122.0, 325.9)	−43.8 (−76.1, −11.5)	**0.001**
HEPTEM MCF (mm)	Intervention I	266	90 days or less	62.2 (58.6, 65.7)	30.1 (26.5, 33.7)	−32.1 (−35.3, −28.8)	**< 0.001**
	Intervention II	205		32.8 (30.1, 35.5)	58.0 (55.3, 60.7)	25.2 (21.5, 29.0)	**< 0.001**
	Intervention III	217		57.0 (53.5, 60.6)	55.2 (51.0, 59.3)	−1.9 (−6.8, 3.1)	1.000
	Intervention IV	188		54.7 (47.6, 61.8)	58.7 (52.0, 65.5)	4.0 (−1.8, 9.9)	0.659
	Intervention I	266	Older than 90 days to 2 years	59.9 (56.4, 63.5)	36.6 (33.1, 40.2)	−23.3 (−25.5, −21.1)	**< 0.001**
	Intervention II	205		38.7 (36.6, 40.8)	56.0 (53.7, 58.4)	17.3 (13.8, 20.9)	**< 0.001**
	Intervention III	217		55.0 (51.8, 58.3)	55.6 (52.2, 59.0)	0.6 (−2.5, 3.7)	1.000
	Intervention IV	188		56.4 (49.6, 63.2)	58.3 (50.9, 65.7)	1.9 (−3.8, 7.6)	1.000
	Intervention I	266	Older than 2 years	59.7 (56.3, 63.1)	47.0 (42.4, 51.6)	−12.7 (−18.3, −7.2)	**< 0.001**
	Intervention II	205		44.3 (40.6, 48.0)	51.0 (40.6, 61.4)	6.7 (−10.4, 23.8)	1.000
	Intervention III	217		52.6 (45.2, 60.0)	50.4 (46.6, 54.2)	−2.2 (−12.1, 7.8)	1.000
	Intervention IV	188		53.2 (45.5, 61.0)	57.1 (49.3, 64.8)	3.9 (−0.9, 8.6)	0.250
FIBTEM MCF (mm)	Intervention I	255	90 days or less	17.0 (13.2, 20.8)	3.7 (0.3, 7.0)	−13.3 (− 15.9, − 10.7)	**< 0.001**
	Intervention II	196		4.3 (3.4, 5.3)	8.5 (7.2, 9.8)	4.2 (2.6, 5.7)	**< 0.001**
	Intervention III	219		8.2 (6.7, 9.7)	10.6 (9.1, 12.1)	2.4 (0.9, 3.9)	**< 0.001**
	Intervention IV	190		10.4 (8.8, 12.0)	12.5 (10.8, 14.2)	2.1 (−0.7, 4.9)	0.396
	Intervention I	255	Older than 90 days to 2 years	15.1 (11.0, 19.2)	4.3 (0.8, 7.7)	−10.9 (−13.1, −8.6)	**< 0.001**
	Intervention II	196		4.6 (3.8, 5.5)	8.3 (7.4, 9.1)	3.6 (2.8, 4.4)	**< 0.001**
	Intervention III	219		7.9 (6.8, 9.0)	10.1 (8.9, 11.3)	2.2 (1.0, 3.4)	**< 0.001**
	Intervention IV	190		10.2 (8.5, 11.9)	11.7 (9.6, 13.8)	1.5 (−0.7, 3.7)	0.736
	Intervention I	255	Older than 2 years	12.8 (9.2, 16.5)	6.8 (3.8, 9.7)	−6.1 (− 8.5, −3.7)	**< 0.001**
	Intervention II	196		6.2 (4.8, 7.6)	7.0 (4.5, 9.5)	0.8 (− 3.8, 5.4)	1.000
	Intervention III	219		7.5 (5.8, 9.3)	10.9 (9.5, 12.3)	3.4 (0.9, 5.9)	**< 0.001**
	Intervention IV	190		11.2 (9.3, 13.1)	9.6 (7.5, 11.6)	−1.6 (−4.3, 1.0)	1.000
HEPTEM α (°)^b^	Intervention I	265	90 days or less	77.0 (73.7, 79.7)	36.6 (31.2, 42.2)	−40.4 (−50.4, −30.5)	**< 0.001**
	Intervention II	204		42.1 (37.8, 46.4)	74.9 (72.1, 77.4)	32.9 (23.2, 42.5)	**< 0.001**
	Intervention III	217		72.8 (69.8, 75.4)	63.9 (60.0, 67.4)	−8.9 (−15.0, −2.8)	**< 0.001**
	Intervention IV	188		64.4 (57.6, 70.3)	70.2 (64.0, 75.2)	5.7 (0.3, 11.2)	**0.038**
	Intervention I	265	Older than 90 days to 2 years	75.0 (71.4, 78.0)	49.5 (43.7, 55.1)	−25.5 (−32.8, −18.3)	**< 0.001**
	Intervention II	204		54.0 (50.5, 57.3)	74.9 (72.4, 77.1)	20.9 (13.9, 28.0)	**< 0.001**
	Intervention III	217		72.4 (69.7, 74.8)	67.9 (64.7, 70.8)	−4.5 (−8.2, −0.8)	**< 0.001**
	Intervention IV	188		67.8 (61.1, 73.3)	68.7 (61.6, 74.5)	0.9 (−1.2, 3.0)	1.000
	Intervention I	265	Older than 2 years	73.4 (69.6, 76.7)	61.3 (55.1, 66.9)	−12.1 (− 19.5, −4.8)	**< 0.001**
	Intervention II	204		57.4 (51.2, 63.1)	69.8 (57.0, 78.6)	12.4 (−3.3, 28.1)	1.000
	Intervention III	217		68.6 (56.9, 77.1)	65.3 (61.9, 68.3)	−3.4 (−9.0, 2.3)	1.000
	Intervention IV	188		66.1 (58.5, 72.4)	69.8 (62.8, 75.4)	3.7 (−1.0, 8.4)	1.000

^a^N was number of observations used for each model. All the models were controlled for age groups, whether each of the interventions was received, as well as interaction between age groups and before or after the intervention

^b^Generalized linear mixed model using beta distribution was applied to predict α (°), while generalized estimation equation was used for other outcomes. Estimates were predicted marginal means

^c^*p* values were Bonferroni-adjusted for estimated mean changes before and after the intervention in each age group

*CI* confidence interval, *CT* clotting time, *MCF* maximum clot firmness

*p* values in bold represent statistical significance

HEPTEM MCF was significantly decreased in all three groups (*p* < 0.001). Compared with patients in the two older age groups, patients ≤90 days had a greater decrease in mean HEPTEM MCF (EMC: -32.1, 95% CI: − 35.3–-28.8). Similarly, FIBTEM MCF and HEPTEM α were significantly reduced in the three age groups, with the youngest group having the greatest decrease (FIBTEM MCF EMC: -13.3, 95% CI: − 15.9–-10.7, *p* < 0.001; HEPTEM α EMC: -40.4, 95% CI: − 50.4–-30.5, *p* < 0.001).

#### Intervention II: PLT administration during the rewarming phase of CPB

Patients in the two younger age groups had a significant decrease in HEPTEM CT (EMCs: − 84.0 and − 72.7, 95% CIs: − 161.1–-7.0 and − 90.8 to − 54.6, respectively, both *p* < 0.001), while no significant change was found in patients > 2 years (*p* = 1.000). HEPTEM MCF rose significantly in the two younger age groups (EMCs: 25.2 and 17.3, 95% CIs: 21.5–29.0 and 13.8–20.9, respectively, both *p* < 0.001), whereas no significant change was seen in patients > 2 years (*p* = 1.000). Consistent results were seen in changes of FIBTEM MCF and HEPTEM α, with the two younger groups demonstrating significant increases (*p* < 0.001), whereas the change in patients > 2 years was not significant (*p* > 1.000).

#### Intervention III: protamine and HFC administration after the termination of CPB

HEPTEM CT increased dramatically in patients ≤90 days old (EMC: 234.9, 95% CI: 89.9–379.8, *p* < 0.001), while it also increased significantly but less notably in patients > 90 days and ≤ 2 years (EMC: 81.0, 95% CI: 51.1–110.9, *p* < 0.001). No significant change in HEPTEM CT was found in patients > 2 years old (*p* = 0.401). HEPTEM MCF did not significantly differ after Intervention III in all three age groups (*p* = 1.000). In contrast, FIBTEM MCF was significantly higher, with an estimated increase of 2.4 mm (95% CI: 0.9–3.9, *p* < 0.001) in patients ≤90 days old, 2.2 mm (95% CI: 1.0–3.4, *p* < 0.001) in patients > 90 days and ≤ 2 years, and 3.4 mm (95% CI: 0.9–5.9, *p* < 0.001) in patients > 2 years old. HEPTEM α decreased significantly in the two younger age groups (*p* < 0.001), while no significant change in HEPTEM α was found in patients > 2 years (*p* = 1.000).

#### Intervention IV: further component therapy if bleeding persists

HEPTEM CT significantly dropped by 152.3 s (EMC: -152.3, 95% CI: − 265.6–-39.0, *p* = 0.001) for patients ≤90 days, whereas it decreased by 43.8 s (EMC: -43.8, 95% CI: − 76.1–-11.5, p = 0.001) for patients > 2 years. The change in HEPTEM CT for patients ≥90 days to ≤2 years was not significantly different from 0 (p = 1.000). No significant change was found in HEPTEM MCF or FIBTEM MCF in all three age groups (*p* > 0.05). HEPTEM α increased by 5.7° (EMC: 5.7, 95% CI: 0.3–11.2, *p* = 0.038) in the youngest patients. There was no significant change in HEPTEM α in patients > 90 days (*p* = 1.000).

## Discussion

The results of this study demonstrate that there are predictable and quantifiable changes in ROTEM values following administration of PLT and HFC during CPB surgery in newborns, neonates and young infants. CPB negatively and significantly impacts all ROTEM values assessed in this analysis (HEPTEM α, HEPTEM CT, HEPTEM MCF, and FIBTEM MCF). The greatest perturbances in coagulation parameters occurred consistently in patients ≤90 days of age, followed by patients > 90 days to ≤2 years of age; while patients older than 2 years were affected the least.

Platelet transfusion (Intervention II) significantly improved all ROTEM parameters. Prolongation of the median HEPTEM CT at timepoints during CBP indicates a deficiency in clotting factors that can be mitigated with the transfusion of FFP. A low HEPTEM α indicates platelet deficiency, and a low FIBTEM MCF indicates fibrinogen deficiency, so improvements in these parameters would be expected. Changes in ROTEM following platelet transfusion can be attributed to clotting factors and fibrinogen in the residual plasma in which the platelets are suspended; this rationale is supported by the observed average increase of 69.8 mg/dL in fibrinogen level.

The normal concentration of fibrinogen in plasma is 160–450 mg/dL [7–9]. HFC is indicated for the treatment of acute bleeding in patients with congenital fibrinogen deficiency [10]. The standard adult dose of HFC is 70 mg/kg when the fibrinogen level is known, but the dose of HFC can be calculated based on actual and target fibrinogen concentrations using the following formula [10]:

$$ \mathrm{Dose}\ \left(\mathrm{mg}/\mathrm{kg}\ \mathrm{body}\ \mathrm{weight}\right)=\frac{\mathrm{target}\ \mathrm{level}\ \left(\mathrm{mg}/\mathrm{dL}\right)\hbox{-} \mathrm{measured}\ \mathrm{level}\ \left(\mathrm{mg}/\mathrm{dL}\right)}{1.7\ \left(\mathrm{mg}/\mathrm{dL}\ \mathrm{per}\ \mathrm{mg}/\mathrm{kg}\ \mathrm{body}\ \mathrm{weight}\right)} $$

In this study, treatment with HFC at a median dose of 70 mg/kg caused an average increase in the FIBTEM MCF of 2.3 mm and an average increase in fibrinogen concentration of 73.1 mg/dL. A pilot study reporting use of a FIBTEM-guided protocol for administration of fibrinogen concentrate to target a high-normal plasma fibrinogen concentration in adult patients undergoing aortic valve operation and ascending aorta replacement demonstrated lower transfusion requirements and lower post-operative bleeding compared with patients receiving conventional transfusion management [11]. However, there is a lack of similar data in pediatric populations. Increased understanding of the typical or expected increases in FIBTEM MCF and fibrinogen concentration with HFC, such as those observed here, would assist clinicians in determining appropriate doses in pediatric patients.

A surprising observation in this study was the prolongation of the HEPTEM CT by 110.8 s and a decrease in the HEPTEM α by 5.6° following HFC administration. Protamine administration is temporally related to HFC administration, and the reversal of heparin and the initiation of thrombus formation would, we suggest, utilize some of the in situ platelets and clotting factors.

All the observed impacts of interventions were magnified in the youngest patient group (≤90 days of age). This would be expected since patients in this cohort would have immature coagulation system [12–14]. There are quantitative and qualitative differences in fibrinogen function between neonates and adults due to the presence of “fetal” fibrinogen [15]. Confocal microscopy has demonstrated that there are significant structural differences between adult and neonatal fibrin networks: neonatal fibrin lacks three-dimensional structure due to the absence of cross-linking, which occurs in adult fibrin networks, making the neonatal clot more porous and less stable [15]. Interestingly, even with treatment with adult fibrinogen, fibrin function is not fully restored [15].

It has previously been shown that use of ROTEM can reduce the need and amount of transfused blood products in pediatric cardiac surgery patients [16]. Additionally, Tirotta et al. have demonstrated that administering HFC at a dose of 70 mg/kg to neonates and infants undergoing cardiac surgery reduced FFP and cryoprecipitate requirements [17]. Targeting a high-normal FIBTEM MCF in this age group may lead to even further reductions in transfusion requirements. Further prospective trials are needed in the pediatric population to test this hypothesis.

## Conclusions

This study demonstrates that CPB profoundly and negatively impacts all ROTEM values in pediatric patients undergoing cardiac surgery. Transfusion with platelets (38 mL/kg) improved HEPTEM α by 22.1° while FIBTEM MCF increased by 2.9 mm. Administration of HFC improved FIBTEM MCF by 2.7 mm and led to an average increase in fibrinogen concentration of 73 mg/dL. Further research is necessary to corroborate these results.

## Data Availability

The datasets used and/or analyzed during the current study are available from the corresponding author on reasonable request.

## Abbreviations

CI: Confidence intervals
CPB: Cardiopulmonary bypass
CS: Cell saver
CT: Clotting time
EMC: Estimated mean change
FFP: Fresh frozen plasma
GEE: Generalized estimation equation
HFC: Human fibrinogen concentrate
IRB: Institutional Review Board
MCF: Maximum clot firmness
PB: Phlebotomized blood
PLT: Platelets
POC: Point of care
PRBC: Packed red blood cells
ROTEM: Rotational thromboelastometry
TEG: Thromboelastography
